# Complex genomic rearrangements in the dystrophin gene due to replication-based mechanisms

**DOI:** 10.1002/mgg3.108

**Published:** 2014-09-15

**Authors:** Berivan Baskin, Dimitri J Stavropoulos, Paige A Rebeiro, Jennifer Orr, Martin Li, Leslie Steele, Christian R Marshall, Edmond G Lemire, Kym M Boycott, William Gibson, Peter N Ray

**Affiliations:** 1 Department of Immunology, Genetics and Pathology, The Rudbeck Laboratory, Uppsala UniversityUppsala, Sweden; 2 The Centre for Applied Genomics, The Hospital for Sick ChildrenToronto, Ontario, Canada; 3 Department of Laboratory Medicine and Pathology, University of TorontoToronto, Ontario, Canada; 4 Division of Molecular Genetics, The Hospital for Sick ChildrenToronto, Ontario, Canada; 5 Division of Medical Genetics, Royal University Hospital & University of SaskatchewanSaskatoon, Saskatchewan, Canada; 6 Department of Genetics, Children's Hospital of Eastern OntarioOttawa, Ontario, Canada; 7 Department of Medical Genetics, Child and Family Research Institute, The University of British ColumbiaVancouver, British Columbia, Canada; 8 Department of Molecular Genetics, The University of TorontoToronto, Ontario, Canada

**Keywords:** Duchenne muscular dystrophy, dystrophin, MMRDR, mRNA, rearrangement, replication

## Abstract

Genomic rearrangements such as intragenic deletions and duplications are the most prevalent type of mutations in the dystrophin gene resulting in Duchenne and Becker muscular dystrophy (D/BMD). These copy number variations (CNVs) are nonrecurrent and can result from either nonhomologous end joining (NHEJ) or microhomology-mediated replication-dependent recombination (MMRDR). We characterized five DMD patients with complex genomic rearrangements using a combination of MLPA/mRNA transcript analysis/custom array comparative hybridization arrays (CGH) and breakpoint sequence analysis to investigate the mechanisms for these rearrangements. Two patients had complex rearrangements that involved microhomologies at breakpoints. One patient had a noncontiguous insertion of 89.7 kb chromosome 4 into intron 43 of *DMD* involving three breakpoints with 2–5 bp microhomology at the junctions. A second patient had an inversion of exon 44 flanked by intronic deletions with two breakpoint junctions each showing 2 bp microhomology. The third patient was a female with an inherited deletion of exon 47 in *DMD* on the maternal allele and a de novo noncontiguous duplication of exons 45–49 in *DMD* and *MID1* on the paternal allele. The other two patients harbored complex noncontiguous duplications within the dystrophin gene. We propose a replication-based mechanisms for all five complex *DMD* rearrangements. This study identifies additional underlying mechanisms in DMD, and provides insight into the molecular bases of these genomic rearrangements.

## Introduction

Duchenne muscular dystrophy (DMD, MIM 310200), the most common and severe neuromuscular disease in humans, is caused by mutations in the dystrophin gene (*DMD*, MIM 300377) located on Xp21. The dystrophin gene, consisting of 79 exons, spans 2.3 Mb of genomic sequence and is one of the largest genes in the genome with 11 kb (0.6%) of coding sequence. Compared with other human genes, the mutation rate in the dystrophin gene is high with approximately 1/3 of the mutations resulting in DMD being de novo with the remaining 2/3 inherited (Cagliani et al. 2004). The most prevalent disease-causing mutations in *DMD* are exonic deletions and duplications accounting for approximately 65% and 10% of the pathogenic alterations, respectively. The remaining mutations are mainly nonsense and indel mutations. Deletions and duplications in *DMD* are nonrandom events with deletion hotspots involving exons 45–50 and duplication hotspots involving exons 2–11 (Aartsma-Rus et al. 2006). Although clustered, these deletions and duplications are typically nonrecurrent with different sizes and distinct breakpoints. In contrast to recurrent rearrangements, nonrecurrent events do not usually originate by nonallelic homologous recombination (NAHR) mainly mediated by low-copy repeats (Sen et al. 2006). Instead, nonhomologous end joining (NHEJ) (ligation of double-strand-breaks) is commonly proposed as a mechanism for nonrecurrent intragenic deletions and duplications (Lieber 2008). Supporting evidence for this in DMD has been shown by sequencing of deletion breakpoint junctions in the dystrophin gene in several studies (Nobile et al. 2002; Oshima et al. 2009; Ankala et al. 2012).

The increased use of gene specific high-resolution tiling comparative hybridization arrays (aCGH) in clinical laboratories has enabled the detection of noncontiguous deletions, duplications, and triplications (Lee et al. 2007; Carvalho et al. 2009; Ishmukhametova et al. 2012). These complex genomic rearrangements consist of more than one simple rearrangement, and have two or more breakpoint junctions. Rearrangements such as these have been suggested to occur by microhomology-mediated replication-dependent recombination (MMRDR); a replication-based mechanism that requires microhomology and includes fork stalling and template switching (FoSTeS) (Lee et al. 2007), serial replication slippage (SRS) (Chen et al. 2010), and microhomology-mediated break-induced replication (MMBIR) (Hastings et al. 2009) models. These models suggest that during replication downstream fork switching results in a deletion, whereas switching to an upstream fork results in duplication and repeated switches back and forth result in complex rearrangements such as triplications and inversions. Previous studies involving replication-based models have been used to explain the mechanism of gross rearrangements in genes causing genomic disorders such as Pelizaeus-Merzbacher disease (Lee et al. 2007), Rett syndrome (Carvalho et al. 2009), and CMT1A/HNPP (Zhang et al. 2010).

Complex genomic rearrangements (CGR) in *DMD* are rare but have been demonstrated (White et al. 2006; Zhang et al. 2008; Oshima et al. 2009; Ishmukhametova et al. 2013, 2012). These studies suggest that CGRs in the dystrophin gene are caused by NHEJ and/or replication-based models. However, few cases of CGRs in *DMD* have been described in detail. We therefore investigated mechanisms causing CGR in a series of five DMD patients identified with complex genetic rearrangements in our diagnostic laboratory. To elucidate the mechanism by which these rearrangements occurred, we used a combination of MLPA/mRNA transcript analysis/custom arrayCGH and breakpoint sequence analysis. We were able to demonstrate that all five cases harbored complex rearrangements within the central region of the dystrophin gene involving noncontiguous deletions, duplications, insertions and inversions. Our studies suggest that replication-based mechanisms are involved in generating these complex rearrangements. We propose that complex genomic rearrangements in the dystrophin gene are a result of MMRDR.

## Materials and Method

### Samples

Peripheral blood samples and muscle biopsies from five patients were submitted to the Molecular Diagnostic Laboratory at the The Hospital for Sick Children. Genomic DNA was extracted from blood and total RNA was extracted from muscle tissue biopsies.

### Multiplex ligation-dependent probe amplification analysis

For detection of deletions and duplications in all 79 exons in *DMD* (NM_004006.1) MLPA was performed on all patients as routine diagnostic analysis. The DMD-MLPA reaction kits (SALSA P034/P035) were obtained from MRC-Holland (Amsterdam, the Netherlands; www.mrc-holland.com). MLPA reactions were performed according to the manufacturer's protocol and analyzed using the GeneMarker software (Softgenetics, State College, PA).

### Dystrophin transcript analysis

In patients where no deletion or duplication is found, we sequence each exon and exon/intron boundary to detect point mutations and splicing mutations in *DMD* (NM_004006.1). If this fails to reveal a causative mutation we proceed to mRNA transcript analysis. Dystrophin transcript analysis was performed by analysis of mRNA extracted from muscle a biopsy. RNA was then transcribed into cDNA using Superscript first-strand synthesis system for reverse transcriptase (RT)-PCR kit (Invitrogen, Carlspad, CA) using manufacturer's protocol. Primers amplifying the entire DMD transcript as a series of overlapping fragments were designed. Standard PCR conditions were used. Primer sequences are available on request.

### High-density array CGH

To investigate rearrangements in higher resolution, we analyzed DNA from patients 2, 3, 4 and 5 with a custom designed CytoSure DMD array, 4 × 44K (Oxford Gene Technology, IP, UK). This array consisted of 44,000 60-mer oligonucleotides, with an average probe spacing of 10 bp within the exons and 106 bp within introns. The reactions were performed according to the manufacturer's protocol using 200 ng of purified genomic DNA. The slides were scanned on an Agilent High-Resolution C Scanner (Agilent, Santa Clara, CA) and analyzed using CytoSure Interpret software (Oxford Gene Technology IP, UK).

To determine the parent of origin of the de novo noncontiguous duplication in patient 3, we genotyped the patient and parents with the Illumina Human 660W-Quad BeadChip.

### Mutation breakpoint mapping (long range-PCR)

Long range PCR was performed on patient 2 using a series of overlapping primers designed to span the 16 kb region of intron 49 and the 45 kb region of intron 50. PCR analysis was carried out to identify the breakpoint junctions of the inversion/deletion in the genomic DNA of the patient. Long-range PCR was performed using Platinum high-fidelity *Taq* DNA polymerase with high-fidelity buffer (Invitrogen, Carlspad, CA) under the following reaction conditions: 94°C for 2 min, followed by 35 cycles of 94°C for 30 s, 55°C for 30 s, 68°C for 1 min per kb. Reactions were electrophoresed on 0.8% agarose gels and amplicons ranging in size from ∼1 kb up to 6 kb could be visualized. Any failure to amplify in the regions within introns 49 and 50 indicated that either the entire fragment or at least one primer annealing site was deleted or inverted. In regions that failed to amplify, overlapping primer pairs were designed and used to obtain fragments containing the breakpoint junctions the fragments were subsequently cycle sequenced using standard conditions.

## Results

Five individuals with a clinical history or diagnosis of DMD that underwent routine analysis in our clinical genetic diagnostic laboratory were found to have complex rearrangements of the dystrophin gene. Further investigation using mRNA transcript analysis and/or high- density custom *DMD* aCGH for high-resolution analysis and long-range PCR amplification for breakpoint determination revealed that these patients had complex rearrangements in *DMD*. The complexities of the rearrangements are summarized in Table1.

**Table 1:** Summary of complex dystrophin rearrangements in five DMD patients

Patient	Sex	Complexity of rearrangement	Molecular analysis		Origin of rearrangement	Microhomology at breakpoint junctions	Proposed mechanism of rearrangement
			Analysis	Results			
1	M	nrml-ins1chr4-ins2chr4_nrml	MLPA	Normal	Maternal	jct1: CATATA	MMRDR
			mRNA	ins80bpChr4		jct2: TTTC	
			chr4aCGH	ins1-ins2		jct3: CT	
2	M	del-inv-del	MLPA	Normal	De novo	jct1: CT	MMRDR
			mRNA	del x50		jct2: TG	
			DMD aCGH	del intr49-nrml-del intr50			
3	F	allele1: del	MLPA	dup x45_46-nrml-dup x48_51	Maternal (allele1)	–	NHEJ
		allele2: dup^1^-nrml-dup	DMD aCGH	dup^1^-nrml-dup-nrml-dup	De novo (allele2)	–	MMRDR
4	M	dup-nrml-dup-nrml-dup	MLPA	dup x45_51-nrml-dup x60_67	Maternal	–	MMRDR
			DMD aCGH	dup-nrml-dup-nrml-dup			
5	M	dup-nrml-dup	MLPA DMD aCGH	dup x1-nrml-dup x10_11 dup-nrml-dup	Maternal	–	MMRDR

1 This duplication involves *MID1* located upstream of *DMD*.

### Patient 1: Noncontiguous insertions from chromosome 4 into *DMD*

**Figure 1:**
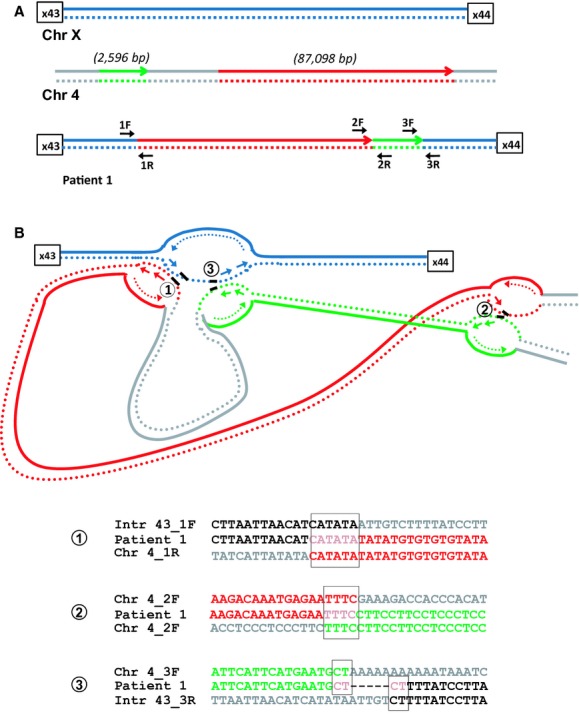
Junction analysis for patient 1 (A) Schematic overview of chromosome 4 insertion in intron 43 of *DMD*. Two segments of chromosome 4, one large (87,098 bp) shown in red and one small (2596 bp) shown in green were inserted into intron 43 of *DMD*. Position of primers for amplification of the three breakpoint junctions are indicated with 1F/1R, 2F/2R, and 3F/3R B). Sequence data alignment for breakpoint junctions (microhomologies are boxed). Six base pairs, four base pairs, and two base pairs of microhomology were found at three breakpoint junctions; (1) proximal *DMD* (1F, blue) and 5′ end of the large chromosome 4 segment (1R, red), 2) 3′ end of the large chromosome 4 segment (2F, red) and 5′ end of the small chromosome 4 segment (2R, green), and 3) 3′ end of the small chromosome 4 segment (3F, green) and distal *DMD* (3R, blue). Bottom panel shows the proposed FoSTeS/MMBIR model for the complex rearrangement. After encountering a DNA lesion (1), the lagging strand (blue, dotted line) in the replication fork of intron 43 would invade a second fork in chromosome 4 (red, dotted line) followed by DNA synthesis. After encountering a second lesion, the newly synthesized lagging strand (2) (red, dotted line) would invade a third replication fork in chromosome 4 (green, dotted line). Resumption of replication on the original template (3) would result in the complex rearrangement seen in the patient.

We previously reported a male patient (pt1: Table1) with an insertion of chromosome 4 sequence into intron 43 of *DMD* (Baskin et al. 2011). This insertion introduced an 80 bp cryptic exon between exons 43 and 44 in the mature *DMD* mRNA resulting in aberrant transcription and the production of no functional dystrophin. The genomic DNA insertion was a complex rearrangement consisting of two noncontiguous segments, one large (87,098 bp) and one small (2596 bp) region of chromosome 4 (Fig.1A). Sequence data alignment of the three breakpoint junctions revealed microhomology (Fig.1B). A six base microhomology sequence (CATATA) was identified at the proximal breakpoint junction of intron 43 in *DMD* and the 5′ end of the large chromosome 4 fragment. A four base pair microhomology sequence (TTTC) was observed at the 3′end of the large chromosome 4 fragment and 5′ end of the small chromosome 4 segment. A two base pair microhomology sequence (CT) was identified at the breakpoint junction of the 3′ end of the small chromosome 4 segment and the distal *DMD*, suggesting a replication-based mechanism for the rearrangement. This rearrangement was inherited from the patient's mother.

### Patient 2: Deletion/Inversion in *DMD*

**Figure 2:**
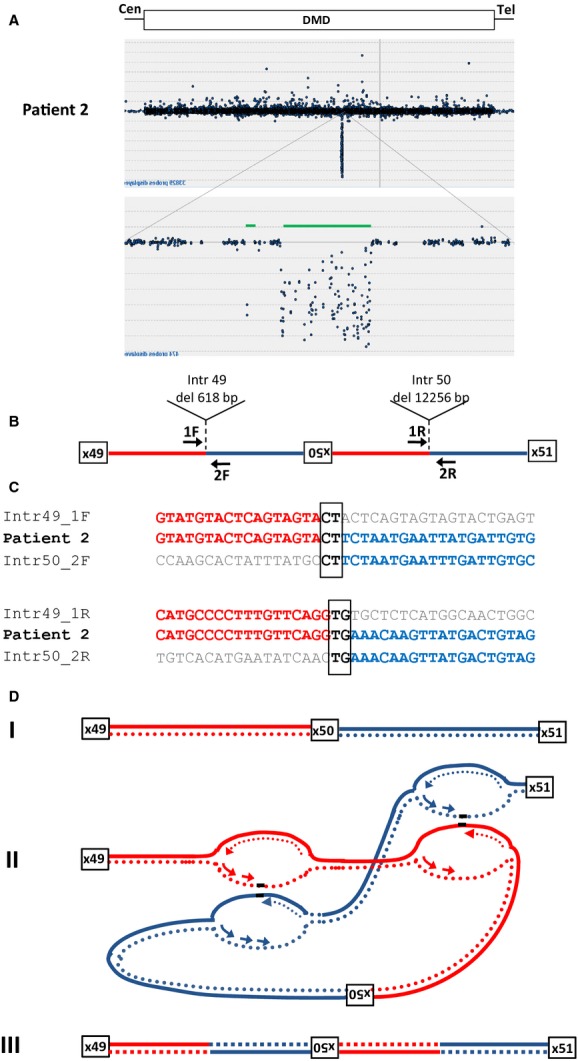
Junction analysis for patient 2 (A) Tiled *DMD* array-CGH profile of patient 2. Genomic DNA from the patient was hybridized to a CytoSure 4 × 44k *DMD* tiling array to determine the size of the chromosome X deletions flanking the inverted exon 50 of *DMD*. (B) The deletions flanking the inversion were 12.2 kb in intron 50 and 618 bp in intron 49, respectively. (C) Alignment of sequences at the junctions. Sequence data alignment for breakpoint junctions (microhomologies are boxed). Two base pairs microhomologies were found at each of the two breakpoint junctions; the reverse complement strand of intron 49 (1F, red) and the inverted complement strand of intron 50 (2F, blue) and the inverted complement strand of intron 49 (1R, red) and the reverse complement strand of intron 50 (2R, blue) are shown. (D) Complex rearrangement explained via the FoSTeS/MMBIR mechanism: (I) A DNA lesion is encountered in the first replication fork (red, solid line) of intron 49; the lagging strand (red, dotted line) disengages and invades the leading strand (blue, dotted line) in the first replication fork (dark blue, solid line) of intron 50 thus facilitating resumption of replication. (2) Simultaneously, another DNA lesion is encountered in a second replication fork (red, solid line) on intron 49 downstream of the initial fork. The leading strand (orange, dotted line) disengages and invades the lagging strand (dark blue, dotted line) of a second replication fork (light blue, solid line) on intron 50 downstream of that original fork. Dotted lines represent newly synthesized DNA. (3) Resumption of replication on the original template occurs.

A male with DMD (patient 2) with no mutation detected in the genomic DNA by either MLPA or sequence analysis of all exons was found upon mRNA transcript analysis to have a deletion of exon 50 in his *DMD* transcript. Sequence analysis of genomic DNA approximately one 1 kb upstream and downstream of exon 50 did not reveal any sequence changes that would result in exon skipping. We performed high-resolution tiling *DMD* CGH array analysis to look for any abnormalities that could explain the skipping of exon 50 in the transcript. Two intronic deletions flanking a region with normal copy number were observed suggesting a potential genetic rearrangement. Long-range PCR amplification and subsequent sequence analysis of breakpoint junctions revealed an inverted 4260 bp segment flanked by a 618 bp deletion in intron 49 and a 12344 bp deletion in intron 50 (Fig.2A). Sequence data alignment of the two breakpoint junctions revealed microhomology (Fig.2B). A two base pair microhomology sequence (CT) was identified at the proximal breakpoint junction of intron 49 and the inverted exon 50 segment. The distal breakpoint also showed a two base pair microhomology sequence (TG) between the inverted segment and intron 50. The rearrangement in this patient was de novo (Table1).

### Patient 3: De novo contiguous duplication and deletion in a female affected with DMD

**Figure 3:**
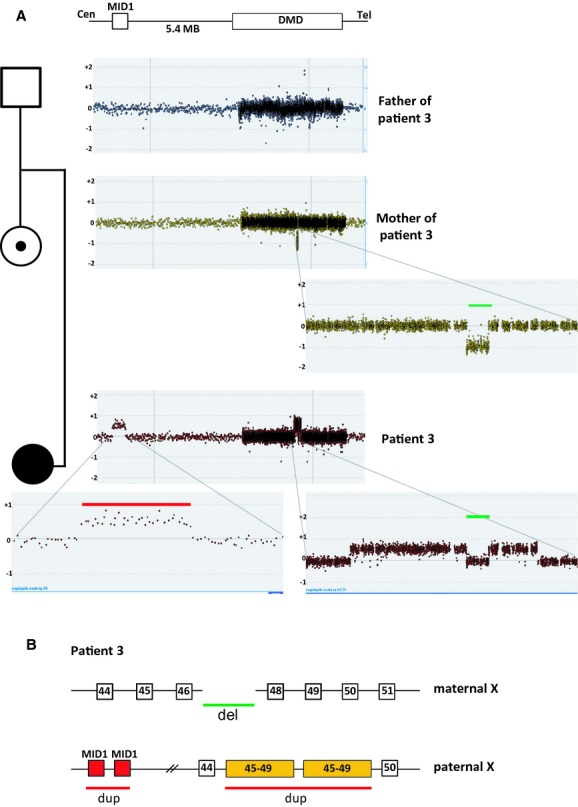
Microarray analysis for patient 3 A) Tiled *DMD* array-CGH profile of patient 3 and her parents. Genomic DNA from patient 3 and her parents were hybridized to a CytoSure 4 × 44k *DMD* tiling array to determine the carrier status in the parents and to confirm the duplication breakpoint junctions in patient 3 on *DMD*. No deletion/duplication was identified in the father, a deletion of exon 47 was revealed in the mother and an apparent duplication, deletion, duplication was seen in the proband. B) Schematic overview of the *DMD* rearrangements in the patient with a deletion of exon 47 on the maternal allele and a de novo non-contiguous duplication (*MID1*, upstream of *DMD* and exons 45–49 of *DMD*) on the paternal allele. Red lines indicate duplications and green lines indicate deletion.

A female DMD patient (patient 3) was found to have duplications of exons 45–46 and 48–51 with normal copy number of exon 47 using MLPA. Carrier testing of her mother revealed that she was a carrier of a deletion of exon 47. To further understand the complexity of the rearrangement identified in the patient, we analyzed the patient's and her parents' genomic DNA on a tiled *DMD* CGH array (Fig.3A). The father of the proband had normal copy number across the dystrophin gene, whereas the mother showed a 30563 bp deletion including exon 47 consistent with the MLPA results. The array CGH data of the patient showed a duplication of 496 kb of *MID1* located 5.4 MB upstream of *DMD* as well as the apparent duplications of exons 45–46 (162 kb) and 48–49 (67.5 kb) with normal dosage of exon 47. The breakpoint junction of the exon 47 deletion in the patient's mother was then sequenced. No microhology was identified at the junction. To establish if the exon 47 deletion was present in the patient, we performed breakpoint junction PCR analysis. The analysis showed the presence of the junction fragment of the exon 47 deletion in the patient (data not shown). This result is consistent with the patient having inherited the exon 47 deletion from her mother and that the noncontiguous duplication seen on the custom array CGH occurred de novo. To determine if the de novo noncontiguous duplication in the proband was on the same allele as the deletion inherited from her mother or if it was on the paternal allele we performed an Illumina 660W SNP array. The SNP array revealed only paternal contribution of probes in the exon 47 region, suggesting that the noncontiguous duplication of *MID1* and exons 45–49 of *DMD* had arisen on the paternal allele. We also performed X-inactivation studies on the proband which revealed random X-inactivation. This further supports the interpretation that the affected female has two disease-causing mutations in trans (one on each allele). Attempts to characterize the duplication junctions with different combinations of long-range PCR amplification were unsuccessful. This suggests further complexity in the rearrangement and that it arose by FoSTeS/MMBIR mechanism.

### Patients 4 and 5: Complex noncontiguous duplications within *DMD*

**Figure 4:**
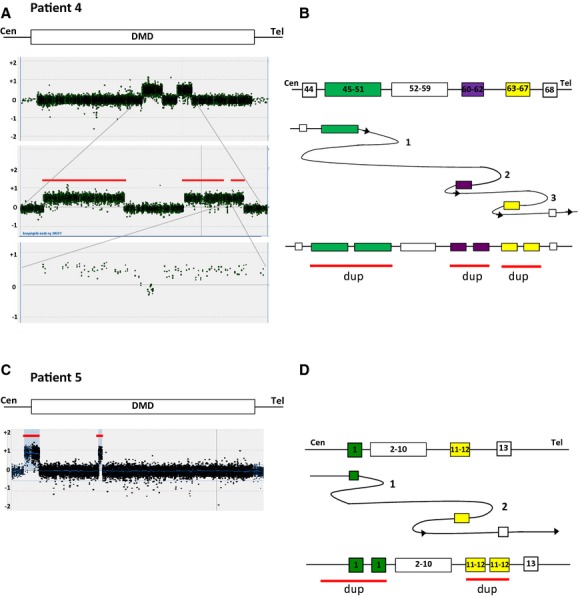
Microarray analysis for patients 4 and 5. Genomic DNA from patients was hybridized to a CytoSure 4 × 44k DMD tiling array to determine the duplication breakpoint junctions on chromosome X of *DMD*. (A) Patient 4: High-density arrays show two duplication segments of the dystophin gene; exons 45–51 and 60–67. The second duplication segment was interrupted with a small region of no copy number changes in intron 62. (C) Patient 5: Two non-contiguous duplications in *DMD* were seen: exon 1 and exons 11–12. (B and D). Schematic overview of the suggested FoSTeS/MMBIR mechanism creating the noncontiguous duplication in *DMD* in patients 4 and 5. The numbers indicate where template switching would have occurred and the arrows indicate the direction. Red lines indicate duplications.

Patient 4, a mother with two affected male offspring was revealed by MLPA to have two noncontiguous duplications of exons 45–51 and exons 60–67. We also analyzed her daughter and found that she carried the same duplications suggesting that they were located on the same allele. By *DMD* tiling aCGH, we determined further complexity (Fig.4A) in the duplication of exons 60–67. This duplication was interrupted by an interval with approximately 350 bp normal copy number between exons 62 and 63. This suggests that the complexity at the rearrangement is: dup ex 45–51/nrml/dup ex 60–62/nrml/dup ex 63–67 (Fig.4B).

Patient 5 was identified to have a noncontiguous duplication of exon 1 and exons 10–11 in *DMD* on routine diagnostics using MLPA. Carrier testing of the mother revealed that she was a carrier of the same duplications as her son. Further analysis of patient 5 by *DMD* tiling aCGH we could see that the duplication of exon 1 started further upstream of the dystrophin gene followed by normal copy number and then duplications of exons 11 and 12 (Fig.4B).

Due to the complexity of the rearrangement breakpoint junctions, further attempts to characterize it at the level of DNA sequence failed on both patients 4 and 5.

## Discussion

Here we have presented evidence to suggest that five independent cases of DMD with CGRs involving the dystrophin gene were generated by replication-based mechanisms. We propose that noncontiguous duplications (3 cases), inversion flanked by deletions (1 case), and a noncontiguous insertion of chromosome 4 (1 case) into the dystrophin gene were caused by MMRDR which includes serial-slippage, FoSTeS, and microhomology-mediated break-induced replication (MMBIR) mechanisms (Chen et al. 2010; Liu et al. 2012).

Complex genomic rearrangements in the dystrophin gene are uncommon and have rarely been characterized in detail. In the studies by Khelifi et al. 2011 and Madden et al. 2009; inversions flanked by deletions in intronic sequence are proposed to be a result of replication-based mechanisms (Madden et al. 2009; Khelifi et al. 2011). In a patient with DMD with a dup-trip/inv-dup Ishmukhametova et al. (2013) described a two-step model where inverted repeats facilitate break-induced replication followed by NHEJ. Other observed CGRs in *DMD* were shown to be noncontiguous duplications and triplications (White et al. 2006; Zhang et al. 2008; Oshima et al. 2009).

We previously reported a DMD case (patient1) with a complex genetic rearrangement involving a complex insertion of chromosome 4 into intron 43 of the dystrophin gene (Baskin et al. 2011). In this study, we characterized this rearrangement in more detail and present evidence to suggest it was caused by a replicative mechanism. We identified microhomology at all three breakpoint junctions (Fig.1B). We propose that after encountering a DNA lesion (1) the lagging strand (blue, dotted line) in the replication fork of intron 43 would invade a second fork in chromosome 4 (red, dotted line) followed by DNA synthesis. After encountering a second lesion, the newly synthesized lagging strand (2) (red, dotted line) would invade a third replication fork in chromosome 4 (green, dotted line). Resumption of replication on the original template (3) would result in the complex rearrangement seen in our patient. This event is consistent with the FoSTes model where fork template switching occurs over long distances (Zhang et al. 2009; Liu et al. 2012).

The inversion of exon 50 flanked by intronic deletions in patient 2 can also be explained by a microhomology-mediated replicative mechanism (Fig.2C). A DNA lesion is encountered in a replication fork of intron 49 where the lagging strand disengages and invades the leading strand in replication fork including intron 50. Simultaneously, another DNA lesion is encountered in a second replication fork on intron 49 downstream of the initial fork. The leading strand disengages and invades the lagging strand of a second replication fork on intron 50 downstream of the original fork. Resumption of replication on the original template would then result in the deletion/inversion observed in this patient. This rearrangement could be explained either by FoSTeS or MMBIR mechanism.

Furthermore, we describe a female DMD patient (patient 3) who was found to have a deletion of exon 47 inherited from her mother. No microhomology at the breakpoint junction suggests NHEJ mechanism for this single exon deletion. On her paternal allele, we detected a de novo noncontiguous duplication of exons 45–49 of *DMD* and *MID1* located upstream of *DMD* (Fig.3). We had no success obtaining the breakpoints despite multiple attempts suggesting further complexity. However, this genetic rearrangement cannot be explained by either NAHR or NHEJ and is most likely the result of a MMRDR. Noncontiguous duplications within the dystrophin gene were also seen in patients 4 and 5. The noncontiguous duplication in patient 4 was even more complex showing duplication of exons 45–51, normal copy numbers of exons 52–59, duplication of exons 60–62, normal copy numbers of intron 62 and duplication of exons 63–67 (Fig.4). This noncontiguous duplication could be explained by a series of replication fork disengaging and lagging strand invasions before resumption of replication on the original fork. The noncontiguous duplication in patient 5 showed duplication of exon 1 followed by normal copy numbers of exons 2–9 and duplication of exons 10–11 and can also be explained by a replicative mechanism.

In summary, we have identified three different types of complex rearrangements in the dystrophin gene that we propose are secondary to replication-based mechanisms; a noncontiguous insertion of chromosome 4 into *DMD,* an intragenic exonic inversion flanked by deletions and noncontiguous duplications.

Complex rearrangements in *DMD* are rare; however, by the use of high-resolution array CGH these events can be more readily revealed and are possibly more common than previously believed. Our findings extend the spectrum of complex events identified in the dystrophin gene.

## Conflict of Interest

None declared.
